# Spermatorrhea with Nocturnal Pollutions

**Published:** 1895-07

**Authors:** 


### Selections and Abstracts.

Dr. Henry Jacobson (*American Medico-Surgical Bulletin*) reaches the following conclusions in the treatment of spermatorrhea with nocturnal pollutions:

First.—Seek other causes than onanism, which, like malaria, are easier to find than the more concealed local diseases of the genitals and rectum. Systematic questioning will often reveal a neuropathic family or personal history; or possibly some of the predisposing constitutional diseases.

Second.—All cases do not improve under the same treatment. Remove the cause, if possible; it can be prevented in some cases by warning the growing youths about the evil after-effects of excessive masturbation.

Third.—Bring the microscope as well as the history of the case to your aid in ascertaining if the case is one of nocturnal pollutions, spermatorrhea, or a combination of both.

Fourth.—Cauterization of the prostatic urethra, either by the negative electrode of the galvanic current, or by a solution of nitrate of silver, in my experience, has more effect in nocturnal pollutions than in spermatorrhoea, unless it proves to be a mixed case. Epididymitis, as a result of cauterization, does not occur as frequently as is claimed by some. I never had this complication in any of my cases; nevertheless, it is best to warn your patient that it may occur.

Fifth.—Weak and anæmic patients need fresh air, tonics, and the best hygienic surroundings more than local treatment. All patients should avoid society as much as possible, and, in fact, excitants of any kind. Indulgence in sexual intercourse should be interdicted or should be indulged in at very rare intervals. Avoid giving them the temporary sexual stimulants. Their diet should be non-stimulating. If you do not possess the cold sound, an ordinary large steel sound may be used, cold, every other night, from five minutes to a half hour at each treatment. Occasionally catarrhal symptoms arise, and then the treatment should be stopped until they subside, under the proper treatment for the complication.

